# Chemistry, Meteorology, and the Function of Digestion, Considered with Reference to Natural Theology

**Published:** 1834-07-01

**Authors:** 


*Chemistry, Meteorology, and the Function of Digestion, considered with reference to Natural Theology.* By WILLIAM PROUT, M.D., F.R.S., Fellow of the Royal College of Physicians.—London, 1834. 8vo. pp. 564.

ONE of the most favourite topics of professed grumblers is the slender encouragement given to literature. Observe, cry the croakers, how a manufacturer of carpets or candelabras rolls in wealth, and a plodding attorney is perfect in the art of transmuting parchment into gold, while a Milton is rewarded for a "Paradise Lost" with ten pounds. Now, among other important differences between the material products of the loom or workshop, and the airy creations of genius, there is one which the pessimists seem to have forgotten. The former depend entirely on patronage; the supply, as the economists say, is proportioned to the demand; and if the ever increasing luxury of the age should require an army of attornies instead of a few regiments, they could be produced with the facility, and almost with the rapidity of mushrooms. The products of genius, on the contrary, it is equally difficult to stifle by indifference, or to encourage by rewards: a Tasso or a Milton wrote for immortality, undismayed by poverty and neglect; while, in our own days, we have known an epic poem to run through twenty title-pages in a twelvemonth, and be lauded in every newspaper from Inverness to Dover, though no artifice could prolong its existence for ten years. Pensions and prizes seem to us eminently calculated to depress literature, and as unsuitable to the true genius as the pendulous abdomen of an alderman would be to a pugilist. Among the disadvantages entailed upon the prize and pension system, (the hothouse for forcing geniuses,) there is one too glaring to be overlooked. The author, instead of writing from his own heart, and imparting to us the rich overflowings of his fancy, is perpetually cribbed and confined by the painful consciousness that he is writing for a coterie of patrons; and the consequence is, that a prize essay may be recognized by its lugubrious stiffness, as different from the flow of a genuine book as a court-lady of the eighteenth century, in hoop, wig, and powder, was from a Greek nymph, "a daughter of ægis-bearing Jove."

Now we apprehend that it was with the intention of demonstrating this great literary truth that the Earl of Bridgewater left his eccentric legacy. To make the matter as plain as possible, he set his legatees to write against Paley, one of the clearest heads who have ever adorned the English church. The great author of the Natural Theology was writing on a favourite subject, and at his leisure; he had the lucid, vigorous and argumentative style of a man of brilliant talents long practised in composition, while his playfulness (unlike the caustic sarcasms of sterner writers,) lends an unexpected charm to the subject, like the lambent flame flickering around the temples of the young Ascanius,—irradiating without consuming. Such a contest can end only in one manner, yet glory may be obtained even by the vanquished; and, if Paley could witness the strife, he might say of each learned legatee, with the sturdy Ajax, in the Metamorphoses, *si non vincet, mecum certâsse feretur.*

As this article must necessarily be confined within very scanty limits, we shall pass over the first book, on Chemistry, which is certainly inferior to the other two, and content ourselves with a few extracts from the treatises on Meteorology and Digestion. The following passage is a good specimen of Dr. Prout's clear and simple style:

"*Of the Propagation of Heat and Light below the Earth's Surface in Water.* Water is a very imperfect conductor of heat in the usual acceptation of the term. Thus, almost any degree of heat may be applied, for a considerable time, to the upper surface of a mass of water, without materially influencing the temperature below; so imperfectly and slowly is heat conducted through this fluid. The process by which heat is communicated through water we have termed convection. When heat is applied to the bottom of a vessel full of this fluid, the portion of the water first heated expands in bulk, and thus becomes specifically lighter; it then rises to the top, carrying with it the newly acquired temperature, while another cold portion, sinking to the bottom, is heated in turn, and so on, till the whole mass becomes uniformly heated.

"With respect to the propagation of light through water, it has been calculated that not a tenth part of the incident light can advance five fathoms downwards in the most translucent water; that even of vertical rays one half is lost in the first seventeen feet, and that they become reduced to one fourth by traversing thirty-four feet, which correspond to the mass of an atmosphere. It thus follows, that only the hundred thousandth part of the vertical rays can penetrate below forty-seven fathoms; which is scarcely equal to the glimmer of twilight; and that the depths of the ocean must be always in perpetual darkness.*

"Such are the general principles by which heat and light are propagated in water. But, in speaking of this fluid, in a former chapter, we alluded to one of the physical properties of water, of the utmost importance in the economy of nature, and which, perhaps, almost more than anything else, indicates design; since, like the composition of the atmosphere, this property of water constitutes an exception, as it were, to a general law, expressly directed to a particular object. We have mentioned that it is a general law, that all bodies, in every state of aggregation, expand by heat and contract by cold; now water forms a marked exception to this law. Like other bodies, water continues to contract on the removal of heat, till its temperature comes down to within a certain distance (7° or 8°) from its freezing point. At this distance water begins again to expand, and the expansion continues till it becomes ice; at which moment of freezing, a sudden and considerable expansion takes place. Hence, the specific gravity of ice is decidedly less than that of water, and the solid necessarily swims on the surface of the fluid. The importance of this anomalous property of water is so great, that it is doubtful whether the present order of nature could have existed without it, even although everything else in the world had remained the same. For instance, were it not for the comparative lightness of ice, this solid, instead of beginning to be formed at the surface of water, would have begun to be formed at the bottom; as the colder water from its greater specific gravity would naturally have sunk: for similar reasons, also, the lowest stratum of ice would have been the last to have melted. Now, let us reflect for a moment upon the consequences of such an arrangement. In the northern, and indeed even in temperate climates, the bottoms of all lakes and deep waters would have been a mass of ice, and totally inaccessible, therefore, to organized beings. During the summer a few feet of the upper part of the ice would, perhaps, have been melted; but what little had thus become melted in summer would again have become solid during winter, and, as the accumulations of ice would have been constant, all the seas, even perhaps to the tropical climates, at least at their bottom, would, long before this time, have been a mass of ice! But what in reality happens? In consequence of the above anomalous properties of water this mischief is entirely prevented, and not a particle of ice can be formed in a lake, or other collection of water, till the whole mass is cooled down to the temperature of 40°, at which temperature the specific gravity of water is at its maximum.

"These properties of water operate in the following manner. On the application of cold to the surface of water, the cooled portion sinks, and its descent forces up a portion of warmer water to the surface, which, after communicating some of its heat to the superincumbent air, sinks in its turn; and this process goes on for

* "Article 'Climate,' in the Encyclopædia Britannica."

a greater or less time, according to the depth of the water. If the depth be not very considerable, the whole body of the water becomes cooled down to 40°; at which temperature the specific gravity not increasing, the circulation ceases, and the *surface* of the water, not the *bottom*, becomes at length so far cooled as to be covered with ice. If the depth of the water be considerable, the application of cold may be long continued without the result of freezing: hence, in this and in other countries not intensely cold, it often happens that deep lakes remain unfrozen during the coldest winters." (P. 246.)

Though we agree with our author in his admiration of this curious provision for preventing the rapid freezing of water, still we cannot allow that, were water destitute of this property, the evil consequences which he supposes must necessarily arise. There are generally half a dozen ways of solving a mathematical problem, and are we to suppose that the Architect of the Universe has but one?

Perhaps, on the whole, the third part of this work, in which Dr. Prout treats of the function of Digestion, is the most interesting. He observes, that oil, sugar, and albumen, are the three great principles of which all organized bodies are essentially constituted; and, though some animals can live for a short time on one alone, yet, for perfect nutrition, a combination of two, if not of all three principles, is absolutely required. Now milk contains all three, and all the most subtle refinements of cookery can produce nothing but disguised imitations of this "great alimentary prototype." The operations of the stomach, according to Dr. Prout, are three.

The first consists in reducing the food to a semifluid state. "This operation seems to be altogether chemical, and is probably effected by *reducing* the properties of these alimentary substances." (P. 488.) Dr. Prout has previously explained the sense in which he uses *reduction:* he means the conversion of a strong into a weak substance; and by a strong compound he means "that its constituent supermolecules are, like those of strong cane-sugar, less complicated than the supermolecules of a *weak* principle, like those of the sugar of honey." (P. 487.)

Secondly. The stomach can change one of the simple alimentary principles into another.

Thirdly. It has the power, within certain limits, of *organizing* and *vitalizing* alimentary substances. "It is impossible to imagine that this organizing agency of the stomach can be chemical; this agency is *vital*, and its nature is completely unknown." (P. 489.)

In his account of the reducing powers of the stomach, our author says,

"Regarding the intimate nature of the agency by which the combination of alimentary substances with water is effected in the stomach, we cannot be said to possess much certain knowledge. This combination appears to be chiefly owing to the agency of a fluid secreted by the stomach, the glands for the formation of which fluid are most numerous toward the pyloric orifice. The aliment having been previously broken down by mastication, and having received an admixture of saliva and of other fluids, is brought into contact with the fluid secreted by the stomach; by which secretion, or by some other energy there in operation, the food that has been introduced into the stomach is associated with water, and thus becomes itself more or less a fluid. Of this important secretion of the stomach, chlorine, in some state or other of combination, is an ingredient—it would seem a necessary ingredient; for the secretion, in its healthy state, always contains more or less of chlorine, the powerful influence of which elementary principle seems mainly to contribute towards effecting the union of the food with water. The chlorine, thus so indispensable to the reducing process, is perhaps more frequently the subject of derangement than anything concerned with the assimilation of the food. It often happens that, instead of chlorine, or a little free muriatic acid, a large quantity of free muriatic acid is elicited; which not only gives rise to much secondary uneasiness, but more or less retards the process of reduction itself. The source of this chlorine or muriatic acid must be the common salt which exists in the blood; to suppose that it is generated, is quite an unnecessary hypothesis. The chlorine is therefore secreted from the blood; and it may be demanded, what is the nature of the agency capable of separating that element from a fluid so heterogeneous as the blood? We are acquainted with one agent that exerts such a power, namely, electricity; and this agent, as we formerly observed, seems to be employed by the animal economy for its operations, in the same manner, and on the same principles, as the materials themselves are employed from which the animal body is constructed. Perhaps, therefore, the decomposition of the salt of the blood may be fairly referred to the immediate agency of this principle, electricity. But here the question arises, what becomes of the soda from which the muriatic acid has been disunited? The soda remains behind, of course, in the blood, and a portion of it, no doubt, is requisite to preserve the weak alkaline condition essential to the fluidity of the blood. But the larger part of this soda is probably directed to the liver, and is elicited with the bile in the duodenum, where it is thus again brought into union with the acid that had been separated from the blood by the stomach. These observations, illustrating the importance of common salt in the animal economy, seem to explain in a satisfactory manner that instinctive craving after this substance which is shewn by all animals.

"Admitting that the decomposition of the salt of the blood is owing to the immediate agency of galvanism, we have, in the principal digestive organs, a kind of galvanic apparatus, of which the mucous membrane of the stomach, and perhaps that of the intestinal canal generally, may be considered as the acid or positive pole; while the hepatic system may, on the same view, be considered as the alkaline or negative pole. Whether such galvanic action be admitted or not, (and the admission is of no very great importance,) what we have above stated may be received as a simple expression of the facts, in so far as they relate to the saline constituents of the blood. Moreover, be the nature of the energies what they may by which these changes are effected, along with these changes, and probably by the aid of the same energies, other very important changes or processes are carried on, to some of which we shall presently have occasion to allude. In the mean time we may close this section by observing, that there is strong reason to believe that the solvent power, which we have described, or some power having a great resemblance to it, exists not only in the stomach, but in every part of an animal body. In all animals there are minute tubes, called absorbents, which originate in every part of their bodies, and, at length uniting, enter the sanguiferous system along with the chyle. Now, the office of these tubes is to remove all those portions of the animal frame which, after having performed their several functions, require to be withdrawn. Of course, before solid parts can be thus removed, they must be dissolved, (*digested*, in fact;) and such solution, in many instances, is probably effected as it is in digestion, by combining these solid parts with water. This supposed analogy between the solvent powers of the stomach and those which must prevail all over the body seems to be strongly confirmed by that similarity of structure and of function existing between the lacteals and the absorbents: they indeed form but one system." (P. 494.)

We agree with our author in supposing that the food becomes a semifluid from its admixture with the gastric juice and saliva; but surely this is inconsistent with his previous supposition, that it depends on the *reduction* of the alimentary substances.

We trust that we shall be acquitted of everything like disrespect towards one of the master-spirits in our profession, when we confess that this work has disappointed us. Our expectations may have been unreasonable, but our author's genius had given them birth; and we are dissatisfied, not because the treatise is defective in matter or manner, but because it wants the author's private mark—the stamp of originality. Yet perhaps several of the observations on digestion may be exempted even from this mitigated censure; and, though this work has some of the diagnostic signs of a bespoken book, it is still the book of Dr. Prout: as Longinus observes, that though the Odyssey bears the marks of old age, it is the old age of Homer.

